# 六氯丁二烯分析方法研究进展

**DOI:** 10.3724/SP.J.1123.2020.05019

**Published:** 2021-01-08

**Authors:** Yaotian WANG, Haiyan ZHANG, Jianbo SHI, Guibin JIANG

**Affiliations:** 1.东北大学理学院化学系, 辽宁 沈阳 110819; 1. Department of Chemistry, College of Sciences, Northeastern University, Shenyang 110819, China; 2.中国科学院生态环境研究中心, 环境化学与生态毒理学国家重点实验室, 北京 100085; 2. State Key Laboratory of Environmental Chemistry and Ecotoxicology, Research Center for Eco-Environmental Sciences, Chinese Academy of Sciences, Beijing 100085, China; 3.浙江工业大学环境学院, 浙江 杭州 310014; 3. College of Environment, Zhejiang University of Technology, Hangzhou 310014, China

**Keywords:** 六氯丁二烯, 持久性有机污染物, 样品前处理, 仪器检测, 综述, hexachlorobutadiene, persistent organic pollutants (POPs), sample pretreatment, instrumental detection, review

## Abstract

六氯丁二烯是一种持久性有机污染物,于2015年和2017年分别被列入《斯德哥尔摩公约》附件A和附件C的受控污染物名单中。六氯丁二烯的来源、环境赋存和影响等研究对控制该新增受控持久性有机污染物污染具有重要意义,而灵敏可靠的六氯丁二烯分析方法是开展相关研究的前提和基础。近年来已有不少学者将六氯丁二烯作为分析目标物之一进行了检测或方法学研究。基于这些研究成果,该文综述了六氯丁二烯分析方法的研究进展,其中重点介绍了空气、水体、土壤、污泥、生物组织等多种介质中六氯丁二烯的样品前处理方法,并比较了各方法的优缺点,以期为该领域的进一步研究提供参考。空气中六氯丁二烯主要由泵抽气通过吸附管而采集,再经热脱附后进行仪器分析,检出限在ng/m^3^水平。也有研究应用聚氨酯泡沫被动采样器和吸附剂填充聚氨酯泡沫被动采样器采集大气中六氯丁二烯及其他污染物。基于吸附剂填充聚氨酯泡沫被动采样器的分析方法灵敏度较高,其对六氯丁二烯的检出限低至0.03 pg/m^3^。然而目前被动采样体积仅根据六氯丁二烯的log *K*_OA_系数估算,未来仍需进一步实验校正。水体样品前处理通常也较简单,通过吹扫捕集、液-液萃取或固相萃取目标物后进行仪器分析。固相萃取法能够同步实现目标物的提取、净化和浓缩,在水样中六氯丁二烯分析方面具有明显优势。固相萃取柱类型以及干燥步骤中柱中残留水分去除率均会影响六氯丁二烯的回收率。灰尘、土壤、沉积物、污泥和生物组织等固体介质样品基质最为复杂,需联合多种方法进行前处理。固体样品中六氯丁二烯提取方法包括索氏提取,加速溶剂萃取和超声萃取,其中超声萃取法应用最为广泛。固体基质净化方面主要采用层析柱色谱法,多根净化柱联用或多层复合柱能够提升净化效果。仪器分析方面,六氯丁二烯主要采用气相色谱和质谱联用检测,高性能质谱检测器如串联质谱能够大大提高六氯丁二烯的检测灵敏度,具有较大的应用潜力。

## Abstract (en)

Hexachlorobutadiene (HCBD) is one of persistent organic pollutants (POPs) listed in Annex A and Annex C of the Stockholm Convention in 2015 and 2017, respectively. Research on the sources, environmental occurrences, and biological effects of HCBD has a great significance in controlling this newly added POPs. Sensitive and credible methods for the determination of HCBD are preconditions and form the basis for related research work. In recent years, many researchers have included HCBD as one of the analytes in monitoring or methodological studies. Based on the results of these studies, this paper reviews the research progress on analytical methods for the determination of HCBD and focuses on sample pretreatment methods for the analysis of HCBD in various matrices such as air, water, soil, sewage sludge, and biological tissues. The advantages and disadvantages of the methods are also compared to provide reference for further research in this field.

For air samples, HCBD was usually collected by passing air through sorbent cartridges. Materials such as Tenax-TA, Carbosieve, Carbopack, Carboxen 1000, or their mixtures were used as the sorbent. HCBD was thermally desorbed and re-concentrated in a trap and finally transferred for instrumental analysis. Limits of detection (LODs) for HCBD in these methods were at the ng/m^3^ scale. Compared to sampling using pumps, passive air samplers (PAS) such as polyurethane foam PAS (PUF-PAS) do not require external power supply and are more convenient for sampling POPs in air at a large scale. The LOD of the sorbent-impregnated PUF PAS (SIP-PAS) method was much lower (0.03 pg/m^3^) than that of the PUF-PAS method (20 pg/m^3^). However, the sampling volumes in the SIP-PAS and PUF-PAS methods (-6 m^3^) calculated from the log *K*_OA_ value of HCBD have significant uncertainty, and this must be confirmed in the future.

For water samples, HCl or copper sulfate was added to the sample immediately after sampling to prevent any biological activities. HCBD can be extracted from water using methods such as the purge and trap method, liquid-liquid extraction (LLE) method, and solid phase extraction (SPE) method. Among these methods, SPE enabled the simultaneous extraction, purification, and concentration of trace HCBD in a single step. Recoveries of HCBD on Strata-X and Envi-Carb SPE cartridges (63%-64%) were higher than those on Envi-disk, Oasis HLB, and Strata-C18 cartridges (31%-46%). Drying is another key step for obtaining high recoveries of HCBD. Disk SPE involving the combination of a high-vacuum pump and a low-humidity atmosphere is an effective way to eliminate the residual water. In addition, a micro SPE method using functionalized polysulfone membranes as sorbents and employing ultrasonic desorption was developed for extracting HCBD from drinking water. The recovery of HCBD reached 102%, with a relative standard deviation (RSD) of 3.5%.

For solid samples such as dust, soil, sediment, sewage sludge, fly ash, and biota tissue, multiple pretreatment methods were used in combination, owing to the more complex matrix. Freeze or air drying, grinding, and sieving of samples were commonly carried out before the extraction. Soxhlet extraction is a typical extraction method for HCBD; however, it requires many organic reagents and is time consuming. The accelerated solvent extraction (ASE) method requires a small amount of organic reagent, and the extraction can be performed rapidly. It was recently applied for the extraction of HCBD from solid samples under 10.34 MPa and at 100 ℃. Purification could be achieved simultaneously by mixing florisil materials with samples in the ASE pool. Nevertheless, employing the ASE methods widely is difficult because of their high costs. Ultrasonic-assisted extraction (UAE) has the same extraction efficiency for HCBD, with much lower costs compared to ASE, and is therefore adopted by most researchers. The type of extraction solvent, solid-to-liquid ratio, ultrasonic temperature, and power affect the extraction efficiency. Ultrasonic extraction at 30 ℃ and 200 W using 30 mL dichloromethane as the extraction solvent resulted in acceptable recoveries (64.0%-69.4%) of HCBD in 2 g fly ash. After extraction, a clean-up step is necessary for the extracts of solid samples. Column chromatography is frequently used for purification. The combined use of several columns or a multilayer column filled with florisil, silica gel, acid silica gel, or alumina can improve the elimination efficiency of interfering substances.

Instrumental analysis for HCBD is mainly performed with a gas chromatograph equipped with a mass spectrometer operating in selected ion monitoring mode. DB-5MS, HP-5MS, HP-1, ZB-5MS, and BP-5 can be used as the chromatographic columns. Qualification ions and quantification ions include *m/z* 225, 223, 260, 227, 190, and 188. GC-MS using an electron ionization (EI) source was more sensitive to HCBD than GC-MS using a positive chemical ionization source (PCI) and atmospheric pressure chemical ionization source (APCI). Gas chromatography-tandem mass spectrometry (GC-MS/MS), gas chromatography-high-resolution mass spectrometry (GC-HRMS), and high-resolution gas chromatography-high-resolution mass spectrometry (HRGC-HRMS) have recently been used for the separation and determination of HCBD and various other organic pollutants. Instrumental detection limits for HCBD in GC-MS/MS, GC-HRMS, and HRGC-HRMS were more than ten times lower than that in GC-MS, indicating the remarkable application potential of these high-performance instruments in HCBD analysis.

六氯丁二烯(hexachlorobutadiene, HCBD)又名1,1,2,3,4,4-六氯-1,3-丁二烯,是一种人工合成的卤代脂肪族化合物。HCBD在工业、农业等领域中用途广泛,如用于铝和石墨棒生产、用作橡胶等聚合物的溶剂、回收含氯气体或从气体中清除其他有机物的清洗剂、传热液体、废核燃料处理时的重稀释剂、电工作业中的隔离液、液压油、润滑油、杀虫剂、除草剂或葡萄园中熏剂^[1,2,3,4]^。《关于持久性有机污染物的斯德哥尔摩公约》(以下简称《公约》)审查委员会确认了HCBD具有持久性、高毒性、生物富集性和潜在长距离迁移能力,并于2015年和2017年将其列入了《公约》附件A和附件C的受控持久性有机污染物(persistent organic pollutants, POPs)名单中^[5,6]^。美国、加拿大和欧洲大部分地区都已停止了HCBD的有意生产。然而,氯化碳氢化合物生产、电解法制镁、塑料和树脂生产、水泥制造等工业以及废物处置(包括历史残留释放)仍在非有意产生和排放HCBD^[7,8]^。目前国内有关HCBD的研究十分有限,其在环境中的赋存和影响仍不清楚,不利于该POPs污染控制。作为公约缔约国,我国面临着繁重的履约工作任务,开展有关HCBD的环境赋存、污染来源、生态影响以及人体暴露危害等研究迫在眉睫。

因具有高挥发性(25 ℃时亨利系数为1044 Pa·m^3^/mol)和疏水性(辛醇-水分配系数为4.78)^[5]^, HCBD会通过挥发、吸附、沉积、生物积累等途径在多种介质中迁移,并从污染源扩散到周边环境乃至偏远地区,最终造成其广泛存在于环境中。氯碱化工厂和有机氯农药厂等点源周边和非点源区域(甚至一些偏远地区)的大气、土壤、动植物以及水体样品中均检测到了HCBD^[9,10,11,12,13,14,15,16,17,18,19,20,21]^,其检出浓度范围分别为0.01 ng/m^3^~1.8 μg/m^3^(大气)、0.03~0.8 μg/L (水体)、0.003~27.9 ng/g干重(土壤)和0.03~24.6 ng/g干重(生物)。尽管在一些环境样品如科罗拉多州西部农村地区大气和法国莱茵河鱼体中HCBD未检出^[22,23,24,25,26,27,28,29,30]^,但这并不代表HCBD不存在,也可能是由于研究使用的分析方法检测灵敏度低(HCBD检出限分别为0.5 ppb(约5.8 μg/m^3^)和10 ng/g干重)造成的。由此可见,灵敏可靠的HCBD分析方法是开展相关研究的必要条件和重要基础。

样品基质复杂程度、提取目标物效率、干扰物去除效果、仪器检测性能均会影响方法的灵敏度、精确度和准确度。近年来已有学者针对不同环境和生物介质开展了HCBD方法学研究或包含HCBD目标物的检测,但尚未有关于其分析方法的系统评述。本文对不同环境介质中HCBD的前处理和仪器分析方法进行了总结,并比较了各方法的优缺点,以期为深入开展HCBD研究提供参考,从中快速选择到合适的方法进行应用或优化方向进行方法改进。

## 1 不同介质样品的前处理方法

样品前处理(包括预处理)不仅可以提取和富集目标物以及去除干扰物,还可以将样品转化为适合保存的状态或适用于仪器分析的形态。这些过程能够直接影响整个分析方法的可靠性和灵敏度,尤其对痕量物质HCBD的检测来说,是不可或缺的重要环节。表1列举了近年来检测目标物包含HCBD的研究采用的样品前处理方法。

**表 1:** 不同介质中六氯丁二烯的样 品前处理方法

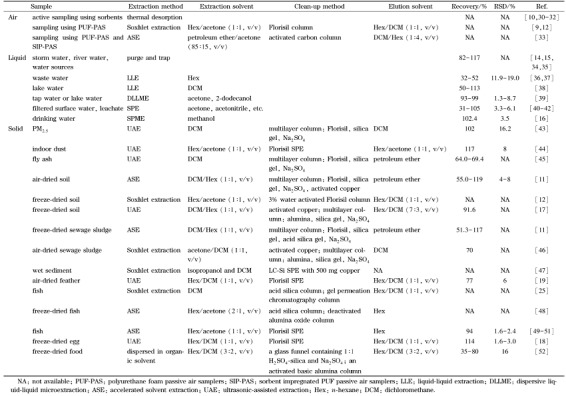

### 1.1 气态介质(室外大气、室内空气)

空气样品中的HCBD主要采用吸附法富集,后续处理与采样器以及吸附材料类型有关。一般利用泵抽气使一定体积的空气通过固体吸附剂,将大气中低浓度的HCBD富集起来,经热脱附后直接进入与热脱附器相连的检测仪器进行分析,HCBD的检出限在几个纳克每立方米到几十纳克每立方米^[10,30-32]^。用于捕集目标物的固体吸附剂可以单独或多种混合使用。例如,使用Tenax-TA和Carbosieve混合吸附剂采集了台湾污染河流上方及沿岸的大气样品中包含HCBD在内的26种挥发性有机化合物(volatile organic compounds, VOCs)^[31]^, Tenax-TA、Carbopack和Carbopack X/B混合材料作为吸附剂采集了西班牙一氯碱厂周边空气中28种物质^[10]^, Tenax-TA、Carboxen 1000和Carbosieve混合吸附剂采集了美国堪萨斯州托儿中心的室内空气中73种VOCs^[30]^。一项方法学研究^[32]^以装有Tenax-TA填料的热脱附管为收集器,对热脱附条件如解吸时间和流速进行了优化以提高23种VOCs分析方法的灵敏度,其中HCBD的检出限和定量限分别降低至0.02和0.08 ng/m^3^。但由于上述研究并非是只针对HCBD这一物质的研究,吸附材料对HCBD的吸附效能以及所需脱附条件可能并未达到最优化,仍需进一步探索以提高HCBD的检测灵敏度。在一些特殊的气态样品如废物填埋以及堆肥散发的恶臭采样时可以先使用采样袋作为采集器收集,再经装有Tenax-TA和Carboxen 1000的吸附管浓缩富集VOCs处理,热脱附后进行仪器检测,HCBD的检出限约几个微克每立方米^[53]^。

大气动力采样需使用不间断电源,在野外采样时难以应用,而且得到的结果通常是短时间内(几十分钟或数小时)的物质浓度^[54]^。相比之下,被动采样器具有不需要额外动力、造价低、易携带等特点,更适合大气中持久性有机污染物的大范围多点位同步采样。两项关于有机氯农药的区域性研究^[9,12]^利用聚氨酯泡沫被动采样器(polyurethane foam passive air samplers, PUF-PAS)分别进行了为期1个月和3个月的大气样品采集,后续PUF样品处理与固体介质类似,经过溶剂萃取以及一定的净化处理。样品中HCBD检出率分别为82%和100%,检出浓度分别在0.03~0.33和0.01~2.23 ng/m^3^范围内。但这两项研究中PUF-PAS对于HCBD的采样体积或速率的校正未见报道,缺乏采样体积或速率校正所得到的浓度结果并不准确^[55]^。另一研究^[33]^基于log *K*_OA_系数估计出PUF-PAS对HCBD的采样体积约6 m^3^。HCBD在PUF上能够很快达到平衡,同时PUF-PAS采集量也会随环境中HCBD量的变化而发生改变,因此PUF-PAS所采集的样品中HCBD仅代表了3个月采样期后期几天的水平。与具有较大收集能力的吸附剂填充PUF被动采样器(sorbent impregnated PUF passive air samplers, SIP-PAS)比较发现,SIP-PAS方法中HCBD的检出限(0.03 pg/m^3^)要远低于PUF-PAS方法(20pg/m^3^),而且SIP-PAS方法采集的HCBD能够代表整个采样期的平均水平^[33]^。然而,基于log *K*_OA_系数计算的HCBD采样速率仍有很大的不确定性,未来还需进一步探索和校正。

### 1.2 液态介质(地表水、地下水、污水等)

目前生物体液中HCBD的研究尚未见报道,液态介质研究对象主要为河水、地下水、污水等环境水样。环境水样采集后需加入稀盐酸降低pH或硫酸铜等以抑制生物活动和防止生物降解,并应尽快进行挥发性有机物的分析,常采用吹扫捕集、液-液萃取(liquid-liquid extraction, LLE)或固相萃取(solid phase extraction, SPE)前处理方法,有时还需过滤膜预处理以去除颗粒物和微生物等。吹扫捕集法比较适合饮用水和水源水等较干净的水体中痕量有机物的检测,HCBD的检出限在1~400 ng/L范围间^[14,15,34,35]^。

LLE法利用待测组分在水相和有机相间分配系数的差异实现组分的提取和分离。针对几百毫升的水样,一般使用几十毫升左右的正己烷、二氯甲烷或石油醚等非极性或弱极性有机溶剂,HCBD的回收率在32%~113%范围间,检出限在零点几个纳克每克到几十纳克每克水平^[36-38,56-58]^。考虑到溶剂环保性,有研究者^[59]^发展了一种使用微量二氯甲烷萃取水中47种VOCs的前处理方法,即向1 mL水样中加入1 mL二氯甲烷并摇匀1 min,加入内标后再加入无水硫酸钠吸水,最后转移出有机相进行检测。该方法有机溶剂用量少,但HCBD的检测灵敏度低,检出限约为1 μg/L,平均回收率为66%。分散液-液微萃取法(dispersive liquid-liquid microextraction, DLLME)是由LLE衍变而来的新方法,使用少量有机溶剂,额外加入的分散剂能够使样品和萃取剂间的接触面积增大,从而提高了萃取效率,缩短了实验时间。例如,以10 μL 2-十二烷醇作为萃取剂和500 μL丙酮作为分散剂快速注入5 mL水样中,待目标物在两相间达到分配平衡后,离心、冰浴、分离出固化的有机相,在室温下融化后进入仪器检测。此方法环保且高效,HCBD的检出限能够达到0.003 μg/L, RSD小于10%,回收率在93%以上^[39]^。

SPE法可同步实现待测组分的提取、纯化和浓缩,更适用于痕量物质的样品前处理。根据目标物的理化性质,选择SPE的填料以及洗脱溶剂类型。在一项关于地表水中36种痕量物质检测方法学研究^[40]^中,加标水平为100 ng/L时不同填料的商品化SPE柱对HCBD的回收率依次为:Strata-X(64%)> Envi-Carb(63%)> Envi-disk(46%)> Oasis HLB(45%)> Strata-C18(31%)。然而我们依照该研究建立的方法进行水体中HCBD的分析,发现水样上载通过柱子后,仅15 min的空气干燥不能把残留在柱中的水分完全除去,造成甲醇、异丙醇和乙腈混合洗脱液中含有微量的水分,可能导致后续氮吹浓缩转换成甲苯溶剂过程中HCBD损失。不同干燥方式对水体中21种有机氯农药SPE法处理效果的影响研究^[41]^表明,通入干燥气体的同时高真空泵抽气15~20 min,能够显著提高柱中水分的去除率,从而提高目标物的回收率。基于C_18_柱对水中HCBD进行的前处理实验中,丙酮作为洗脱液时HCBD的加标回收率(105%±10%)高于乙酸乙酯(72%±6%)。此外,SPE法并不局限于柱状形式,也可以将固体吸附材料直接投入液体样品中进行混合萃取。一种水中8种有机氯化合物的新型前处理方法^[16]^利用膦酸或膦酸酯修饰的聚丙烯膜作为吸附材料和水样混合搅拌50 min,其后采用有机溶剂超声5 min解吸附并进行仪器检测,发现中性水环境中目标物的萃取效率优于碱性和酸性条件。另外,辛醇对HCBD的解吸附效果最好,其次为甲醇,而甲苯、二甲苯以及正己烷对聚丙烯膜上的HCBD洗脱效果较差。优化条件下HCBD的回收率可达102%, RSD为3.5%,检出限为3.9 ng/L。

### 1.3 半固态或固态介质

半固态以及固态样品不仅种类繁多而且形态各异,样品的性质和均匀程度差别较大,一般会对其进行预处理并妥善保存,例如,土壤和底泥样品经冷冻干燥、研磨过筛后室温或低温保存^[11,17]^;生物组织样品均质化后直接冷冻保存或冻干后冷冻保存^[18,19,60]^。相比气态和液态样品,半固态以及固态样品的基质较为复杂,往往需要多种样品前处理方法联合使用。

首先要用有机溶剂提取样品,使目标物所处形态变为液态,常用的提取方法有索氏提取(soxhlet extraction)法、超声辅助萃取(ultrasonic-assisted extraction, UAE)法和加速溶剂萃取(accelerated solvent extraction, ASE)法。传统的索氏提取法回收率高,但萃取时间长(24 h左右),有机溶剂的消耗量较大(100~300 mL),易对环境造成二次污染^[12,25,46,47,61]^。ASE法在较高的压力(10.34 MPa)和温度(100 ℃)下进行目标物提取,相对索氏提取法使用溶剂少,所需时间短,自动化程度高^[11,48]^。一项关于土壤和污泥样品中HCBD和氯苯物质赋存的调查^[11]^采用ASE法提取目标物,在10.34 MPa(1500 psi)和100 ℃条件下,萃取溶剂为1:1(体积比)的二氯甲烷和正己烷混合溶液,静态时间10 min,静置循环次数2次,萃取液经柱净化后检测,HCBD的回收率在51.3%~119%范围间。一项调查苏格兰鳗鱼中持久性有机物赋存水平的研究^[48]^同样采用10.34 MPa和100 ℃条件下ASE法,萃取溶剂为1:2(体积比)的丙酮和正己烷混合溶液,静置时间5 min,静置循环次数5次。另一项鱼体中HCBD和六氯苯分析的方法学研究^[50]^提高了ASE压力,采用13.34 MPa、100 ℃、静态时间10 min和静置循环次数3次条件,萃取溶剂为1:1(体积比)的丙酮和正己烷混合溶液,经柱净化后检测,该方法HCBD的回收率达94%。还有研究^[62]^将弗罗里硅土填料直接置于样品萃取池中,建立了ASE在线净化土壤中多氯烃类化合物的前处理方法,其中HCBD的回收率在76.1%~81.5%之间。尽管ASE法具有较多优点,但仪器使用过程中容易出现填料阻塞管道等故障,其设备和配件成本以及维修费用较高,目前尚难普及。

UAE法萃取速度快,还可以同时处理多组样品,而且仪器占地面积小,仪器及运行成本相对ASE法较低,有机溶剂用量和消耗时间相对索提法较少,操作也比较简单,因此应用十分广泛。从表1中可以看到,针对半固体和固体中包含HCBD的有机物大部分分析研究都采用了UAE法,萃取溶剂一般为二氯甲烷、正己烷、丙酮或其中两种的混合液,处理不大于2 g的样品所需萃取溶剂用量为10~30 mL,总萃取时间在30 min左右,分2到3次萃取^[18,19,43-45]^。根据目标物的物化性质以及样品基质的不同,最优的超声条件也会稍有不同。一项针对飞灰介质中HCBD和五氯苯及六氯苯的方法学研究^[45]^优化了超声条件,在30 ℃和超声功率200 W条件下,二氯甲烷为萃取液、固液比15 mL/g、超声两次、每次15 min即可获得较好的HCBD回收率(64.0%~69.4%)。然而超声温度实际上比较难于精确控制,这是由于超声会使水温上升,超声时间过长会导致水温超过设置温度,而过高的温度可能会导致一定量的HCBD从有机溶剂中挥发,并不利于HCBD的回收分析。另外,飞灰经酸处理后水分较难完全去除,HCBD回收率没有明显提高,若要完全干燥所需时间较长,且容易引入空白污染,因此该方法中并未包含酸处理。此外,超声萃取条件也要随着样品用量的变化而变化。对于10 g冷干后的土壤以及植物样品中的有机氯农药和HCBD,采用1:1(体积比)的正己烷和二氯甲烷混合溶剂超声萃取两次(30 min/次),每次萃取液用量30 mL(共60 mL),经柱净化后仪器检测,HCBD的回收率可达78.2%^[13,17]^。

半固体或固体样品经溶剂萃取目标物后,往往还需要进一步净化去除或降低干扰物影响,减小基体效应,提高检测灵敏度,并防止色谱柱和离子源等仪器设备遭受污染。常用的柱层析色谱法净化样品中HCBD的主要机理包括吸附、分配和体积排阻,使用的固定相填料主要为硅胶、酸性硅胶、弗罗里硅土、氧化铝和凝胶^[11,17,25,44,57]^。部分土壤、河流底泥以及污水处理厂污泥样品处理时还需要加入活化铜粉去硫^[11,17,46,47]^。自制的层析柱一般使用0.9~1.5 cm内径的玻璃柱,填料种类和使用量(几克到十几克)灵活多变^[9,17,43,45,46,48]^。在提升复杂基体净化效果时可以联合使用多根不同填料的层析柱。一项关于鱼组织中多种优先污染物的研究^[25]^使样品萃取液先后经过一根酸性硅胶柱除脂和一根凝胶渗透色谱(gel permeation chromatography, GPC)柱进行净化,其中HCBD的检出限为0.2 ng/g湿重。另一项关于鱼组织中污染物研究^[48]^则联合了一根酸性硅胶柱和一根9%水去活化氧化铝柱净化样品,经检测,HCBD的检出限为1.0 ng/g湿重。多层填料的复合柱应用也较广泛,如弗罗里硅土、硅胶和无水硫酸钠复合柱用于PM_2.5_样品的净化处理^[43]^,氧化铝、硅胶和无水硫酸钠复合柱净化土壤以及动植物样品^[17]^,活化铜粉、弗罗里硅土、活化硅胶、酸性硅胶和无水硫酸钠填制的多层复合柱净化污水处理厂的污泥样品^[11]^。不同填料对飞灰萃取液的净化效果比较结果表明^[45]^,氧化铝和弗罗里硅土净化效果类似,相比硅胶和弗罗里硅土复合柱,单一弗罗里硅土填料柱净化效率较差,可能是由于弗罗里硅土颗粒较大,样品萃取液或有机洗脱液经过柱流速太快,降低了吸附或脱附作用。不同有机溶剂对目标物的洗脱效率也有差别,石油醚作为洗脱剂时HCBD的回收率(93%)高于正己烷及不同比例的正己烷和二氯甲烷的混合液洗脱效率(75%~83%)。该研究^[45]^还发现超声萃取时采用塑料材质的容器会引入杂质干扰、增大基体效应,且不能通过层析柱去除,建议使用玻璃容器。

柱层析色谱法形式不局限于玻璃柱色谱。一项针对肉类、蛋类和蔬菜等食物样品中多种POPs的研究^[52]^将样品直接分散在50 mL 3:2(体积比)的正己烷和二氯甲烷混合溶液中,再向其中加入30 g酸性硅胶和2 mL壬烷,这些混合物随后通过盛有酸性硅胶和无水硫酸的玻璃漏斗过滤,并经3:2(体积比)的正己烷和二氯甲烷混合溶液洗脱,最终液体浓缩后进入仪器检测,HCBD的方法检出限在0.01~0.03 ng/g湿重范围内。此外,SPE小柱也被用于半固体或固体样品的净化处理。一些研究者直接购买商品化的弗罗里硅土SPE小柱或正相硅胶SPE小柱应用于室内灰尘、河流沉积物、飞禽羽毛或鸡蛋冷干粉的样品前处理^[18,19,44,47]^。也有研究者将15 g弗罗里硅土和10 g无水硫酸钠填入聚乙烯材质管自制SPE小柱用于鱼组织萃取液的净化^[50]^。

## 2 仪器检测方法

表2总结了HCBD的常用仪器检测方法。在实际应用中,HCBD常与其他目标化合物一起分析,样品中的待测组分需经气相色谱(GC)分离后进入检测器(电子捕获检测器, ECD)或质谱检测器(MS))检测,最终得到定性定量分析结果。其中,气相色谱多采用非极性或弱极性的毛细管柱,常用的毛细管柱型号包括DB-5MS、DB-624、HP-1、HP-5、HP-5MS、ZB-5MS等^[9,11,12,14,16-18,20,36-38,43-46,52,56]^。

**表 2:** 六氯丁二烯的仪器分析方法

Analytes	Instrumental method	Chromato- graphic column	Monitored ion for HCBD (m/z)	Injection volume/ μL	Instrumental detection limit for HCBD^*^	Ref.		
HCBD, OCPs	GC-ECD	HP-5	NA	1-2	NA			[9,12,17]
HCBD, 13 other compounds	GC-ECD	NA	NA	NA	30 ng/L			[36]
HCBD, CBzs	GC-MS	DB-5MS	225, 260	1	0.001-0.9 μg/m^3^			[45]
HCBD, VOCs	GC-MS	DB-624	NA	NA	NA			[14,34]
HCBD, VOCs	GC-MS	DB-VRX	NA	NA	NA			[35]
HCBD, CBzs	GC-MS	SGE-HT8	225, 223	1	NA			[49-51]
HCBD, 19 other compounds	GC-MS	HP-5MS	225, 260, 227	1-2	NA			[40]
HCBD, CBzs	GC-MS	HP-5MS	225, 260	2	NA			[11]
HCBD, PCBs, OCPs	GC-MS	HP-5MS	260, 225, 190	2	NA			[19]
HCBD, 7 other compounds	GC-MS	HP-1	260	2	3.9 ng/L			[16]
HCBD, semi-VOCs	GC-MS	HP-1	225, 223, 227	1	0.14 ng/g			[46]
HCBD, CBzs, PBDEs	GC-MS	CPSil8	NA	NA	1-6.25 pg/μL			[25]
HCBD, OCPs	PTV-LVI-GC-MS	ZB-5MS	225, 223, 227	100	NA			[41]
HCBD, CBzs	GC-MS	BP-5	225, 223, 227	NA	NA			[47]
HCBD, 5 other compounds	GC-EI-MS	TG-5MS	260	1	1.79 pg/mL			[43]
HCBD, 5 other compounds	GC-EI-MS/MS	TG-5MS	227, 192, 225, 190	1	0.41 pg/mL			[43]
HCBD, 5 other compounds	GC-PCI-MS/MS	TG-5MS	227, 192, 225, 190	1	54.4 pg/mL			[43]
HCBD, 5 other compounds	GC-APCI-MS/MS	TG-5MS	262, 227, 153	1	47.6 pg/mL			[43]
HCBD, 56 other compounds	GC-MS/MS	VF-5MS	224.9, 189.8, 154.9, 152.7	4	0.09 ng/L			[37]
HCBD, 58 other compounds	GC-MS/MS	ZB-5MS	225, 190, 118	2	0.01 pg			[44]
HCBD, OCPs, PCBs	GC-MS/MS	DB-5MS	224.8, 222.8, 189.9, 187.8	1	0.042 pg/μL			[33]
HCBD, OCPs	GC-MS/MS	HP-5MS	225, 260, 190	2	0.02 pg			[18]
HCBD, 13 other compounds	GC-HRMS	ZB-5MS	224.844, 222.841	1	0.225 ng/L			[36]
HCBD, OCPs, PCBz, CBzs, PCP	HRGC-HRMS	DB-5MS	224.8413, 222.8443	1-10	NA			[38,52]

CBzs: chlorobenzenes; OCPs: organochlorine pesticides; PCBs: polychlorinated biphenyls; VOCs: volatile organic compounds; PCP: pentachlorophenol. NA: not available; ECD: electron capture detector; PTV-LVI: programmable temperature vaporizer-large-volume injection; EI: electron ionization source; PCI: positive chemical ionization source; APCI: atmospheric pressure chemical ionization source. * Instrument detection limit (IDL),the smallest value of the analyte detected by an analytical instrument, is generally estimated based on a signal-to-noise ratio of 3.

### 2.1 气相色谱-电子捕获检测

电子捕获检测器是气相色谱仪配备的检测器之一,其对电负性物质具有较高响应,适用于分析卤代有机化合物。一项关于水中有机物分析方法的研究^[39]^采用GC-ECD检测HCBD的方法检出限(0.003 μg/L)约为采用GC-MS检测时检出限的1/10 (0.045 μg/L)。GC-ECD仪器及运行费用低,应用较为普遍,但无法去除各组分间的相互干扰,也不能对共流出物进行准确的定性分析。因此有研究结合GC-MS和GC-ECD分别对HCBD在内的21种有机氯农药进行了定性和定量分析,该方法在保证定性分析准确的前提下,兼顾了定量分析的高灵敏度检测^[12]^。

### 2.2 气相色谱-质谱检测

GC-MS法能够在一定程度上排除干扰物的影响,定性定量分析结果更为准确,因此大部分研究采用了GC-MS分析HCBD和其他有机物(见表2)。离子源是质谱仪的关键部位,在同等条件下比较不同类型离子源对HCBD检测的影响,发现电子轰击源(electron ionization source, EI)较正化学电离源(positive chemical ionization source, PCI)以及大气压化学电离源(atmospheric pressure chemical ionization source, APCI)具有更高的HCBD检测灵敏度^[43]^。目前质谱检测HCBD主要采用EI源,在选择离子扫描模式下,通过若干个碎片离子对其进行定性和定量。常用的HCBD特征离子有225、223、260、190、188等^[11,16,20,33,45,50]^。使用^13^C同位素标记的HCBD(^13^C_4_-HCBD)作为内标时,其定量和定性离子设为231和233, HCBD的定量和定性离子设为225和223^[50]^。但值得注意的是,^13^C_4_-HCBD的碎片离子也有225,虽然强度较低,但若加入内标浓度过高可能会影响样品中痕量HCBD的判断。一般采用不分流进样模式,进样体积为1~10 μL。程序升温大体积进样(programmable temperature vaporizer-large-volume injection, PTV-LVI)能够提高痕量有机物质分析的灵敏度^[63]^。采用100 μL进样体积的PTV-LVI-GC-MS检测水中有机氯时,HCBD的定量限(信噪比9)可低至0.02 ng/L^[41]^。

近年来高性能检测器发展迅速,气相色谱-串联质谱(gas chromatography-tandem mass spectrometry, GC-MS/MS)和气相色谱-高分辨质谱(gas chromatography high-resolution mass spectrometry, GC-HRMS)的应用为复杂基体中痕量HCBD的检测提供了新方法,能够显著提高HCBD检测的灵敏度和准确性。一项关于鸟卵中HCBD的分析研究^[18]^表明,GC-MS/MS对HCBD的仪器检出限比GC-MS方法低一个数量级以上,低至0.02 pg。另两项关于西班牙污水处理厂排出废水中的多种有机污染物研究^[36,37]^应用不同检测器也得到了类似的结论,GC-MS/MS对HCBD的检测灵敏度最佳(0.09 ng/L),其次为GC-HRMS(0.225 ng/L),这两种检测器均优于GC-ECD(30 ng/L)。另外,跟传统气相色谱柱相比,高分辨气相色谱(high-resolution gas chromatography, HRGC)柱固定相液固定在柱管内壁而形成空心柱,通气性较好,能够使得分离效率提高。水体中9种多溴联苯醚、7种多氯联苯和20种有机氯农药(包含HCBD)^[38]^,鱼体中三氯杀螨醇、六溴环十二烷、六氯苯、HCBD、七氯环氧物、多溴联苯醚、多氯联苯和二噁英等优先控制污染物^[28]^,以及食品中HCBD、多氯联苯、六氯苯、五氯苯酚和多氯萘^[52]^均通过HRGC-HRMS得到了分离测定。可见,HRGC-HRMS十分有利于大量复杂有机化合物的分析。

## 3 总结与展望

本文总结了多种介质中HCBD的样品前处理和仪器检测方法及其优缺点。HCBD的样品前处理主要包括:1)固体吸附剂富集空气中目标物后热脱附或溶剂萃取,其中SIP-PAS无需用电、捕集效率较高;2)吹扫捕集法、LLE法或SPE法提取水体中目标物,其中SPE法能够同步实现富集和纯化,但进行洗脱过程前需注意柱中水分的去除;3)索氏提取法、ASE法或UAE法提取半固态及固态样品中目标物后柱层析色谱法净化,多根层析柱联用或多层复合柱去除干扰物质效果较好。HCBD的仪器检测方法为GC分离后ECD或MS检测。

由于HCBD在环境样品中的赋存水平普遍较低,发展高灵敏度和高选择性的HCBD分析方法仍将是未来HCBD研究的重点之一。微尺度材料在污染物的富集和分离方面已表现出明显优势。可根据HCBD的理化性质,设计、制备用于分离富集HCBD的新型微尺度材料,建立HCBD的样品前处理新方法。在仪器检测方面,高性能仪器如GC-MS/MS、GC-HRMS以及HRGC-HRMS能够显著降低HCBD的检出限,在HCBD与其他物质共同分析时具有较大的应用潜力,值得进一步探究。此外,生物体各部分中HCBD的含量和分布是研究HCBD在生物体内代谢转移过程以及毒性效应的关键。除鱼肉组织外,其他生物组织和器官以及血液等介质中HCBD的分析方法尚未建立,这也将成为今后HCBD分析研究的一个方向。
